# Treatment history as early indicators of short-term response to ACTH therapy in infantile epileptic spasms syndrome: a propensity score matching analysis

**DOI:** 10.1186/s42466-026-00513-4

**Published:** 2026-07-14

**Authors:** Kana Lin, Guangyun Zhang, Yiran Zhou, Cuijin Wang, Feng Han, Li Liu, Jie Liu, Changhua Mou, Yunqing Zhou, Jiwen Wang, Dawei Yuan, Yingyan Wang

**Affiliations:** 1 Department of Neurology, Shanghai Children’s Medical Center, Shanghai Jiao Tong University School of Medicine, Shanghai, China; 2 Clinical Research Center, Shanghai Children’s Medical Center, Shanghai Jiao Tong University School of Medicine, Shanghai, China; 3 Pediatric Neurology Department, The First People’s Hospital of Lianyungang, The Affiliated Lianyungang Hospital of Xuzhou Medical University, Lianyungang, China; 4 Department of Neurology, Linyi Maternal and Child Health Hospital, Linyi Branch of Shanghai Children’s Medical Center, Linyi, Shandong Province China

**Keywords:** Infantile Epileptic Spasms Syndrome, ACTH, Short-term response, Treatment history, Propensity score matching

## Abstract

**Background:**

Adrenocorticotropic hormone (ACTH) is a first-line therapy for infantile epileptic spasms syndrome (IESS), along with oral steroids and vigabatrin. the UKISS/ICISS trials have established the superior short-term efficacy of hormonal therapy over vigabatrin and the potential benefit of combination therapy. However, early identification of short-term responders to ACTH remains a clinical challenge. This study investigated whether treatment history, including pre-treatment exposure and on-treatment events, can serve as early indicators of short-term response using propensity score matching (PSM) analysis.

**Methods:**

In this retrospective cohort study, we analyzed IESS patients who received ACTH therapy at Shanghai Children’s Medical Center from March 2015 to February 2024. Responders were defined as those achieving ≥ 50% spasm reduction. PSM was applied using covariates with *P* < 0.20 in pre-matching analysis to balance baseline characteristics between responders and non-responders. Treatment history variables were compared using Chi-square or Fisher’s exact tests. Variables with *P* < 0.20 in univariate analysis were included in a penalized logistic regression with L1 penalty (LASSO) to identify independent indicators.

**Results:**

Among 162 enrolled patients, 110 (55 responders, 55 non-responders) were matched with well-balanced baselines. Multivariate analysis using LASSO regression identified the addition of new antiseizure medications (ASMs) during ACTH therapy (OR = 0.055, 95% CI: 0.012–0.250, *P* < 0.001) and frequent ACTH dose adjustments (one adjustment: OR = 0.153, 95% CI: 0.030–0.780, *P* = 0.024; ≥2 adjustments: OR = 0.251, 95% CI: 0.086–0.731, *P* = 0.012) as having strong associations with lower odds of good short-term response, despite wide confidence intervals. Importantly, these factors likely represent markers of refractory disease rather than independent early predictors. Prior steroid/ACTH use showed a non-significant trend toward higher odds of good response (OR = 3.203, 95% CI: 0.794–12.923, *P* = 0.102).

**Conclusions:**

The addition of new ASMs and frequent dose adjustments during ACTH therapy might reflect early clinical concern for poor response and should prompt reassessment of treatment strategy. Recognizing these signals may facilitate timely clinical intervention.

**Supplementary Information:**

The online version contains supplementary material available at 10.1186/s42466-026-00513-4.

## Background

Infantile epileptic spasms syndrome (IESS) is a severe early-life epileptic encephalopathy characterized by epileptic spasms, hypsarrhythmia on electroencephalography (EEG), and developmental regression or delay [1, 2]. With an estimated incidence of approximately 1 in 2000–4000 live births, IESS represents one of the most challenging epilepsy syndromes in infancy due to its poor prognosis and frequent evolution to Lennox-Gastaut syndrome [3–5]. Without prompt and effective intervention, the condition often leads to drug-resistant epilepsy and irreversible neurological deficits, with only 15–25% of affected children achieving normal neurodevelopmental outcomes [6].

There are three recommended first treatments for IESS: adrenocorticotropic hormone (ACTH), oral corticosteroids, and vigabatrin [7]. Among these, ACTH has reported response rates of 36.7–87% across different studies [7–9]. According to current guidelines, ACTH is considered more effective than vigabatrin in patients without tuberous sclerosis complex [10]. ACTH and prednisolone regimens demonstrated comparable short-term efficacy, while prednisolone exhibited a significant economic advantage [11]. Therapeutic response varies considerably among individuals, and early identification of patients who may not respond adequately remains a significant clinical challenge [12].

Previous studies have identified several factors that may influence treatment response in IESS, including etiology, age at onset, treatment delay, and baseline developmental status [13, 14]. The United Kingdom Infantile Spasms Study (UKISS) and the International Collaborative Infantile Spasms Study (ICISS) demonstrated that hormonal therapy achieves superior short-term spasm control compared to vigabatrin, and that combination therapy may offer additional benefits [13, 15, 16]. There was a statistically significant decline in response rate in those infants who had a lead time to treatment greater than 2 months, and high risk of developmental impairment was the other variable that was significantly associated with the primary outcome [13]. Recent research has further elucidated factors influencing ACTH response, including prior antiseizure medication (ASM) exposure and early EEG changes [17, 18]. A recent study developed a predictive model for initial response to first‑line treatment, identifying key predictors such as age at onset, lead time, and MRI findings [19].

Despite these advances, limited attention has been paid to the prognostic value of a patient’s complete treatment history, which encompasses both pre‑treatment exposure and clinical events during ACTH therapy itself. In this study, we adopt a broad definition of treatment history that includes pre-treatment exposure (prior ASMs, steroids, non-pharmacological interventions) and on-treatment events (medication additions, dose adjustments made by physicians in response to initial therapeutic effect). Understanding these factors as early indicators of response could guide more timely and appropriate treatment modifications [20]. Using real-world data from Shanghai Children’s Medical Center, this study employed propensity score matching (PSM) to balance baseline characteristics and investigate whether treatment history, particularly on‑treatment events, can serve as early indicators of poor short-term response during ACTH therapy in IESS patients.

## Methods

### Study design and population

This retrospective cohort study included patients diagnosed with IESS who received ACTH therapy at Shanghai Children’s Medical Center, Shanghai Jiao Tong University School of Medicine, between March 2015 and February 2024. Data were collected using a standardized electronic case report form. Eligible patients met the following criteria: (1) age 1–24 months at symptom onset; (2) IESS diagnosis confirmed by two pediatric neurologists according to the 2022 ILAE definition [2]; and (3) receipt of ACTH treatment during hospitalization with complete records. Exclusion criteria were: (1) insufficient clinical data; (2) contraindications to ACTH; (3) concomitant tuberous sclerosis complex; (4) evidence of infection prior to ACTH initiation; or (5) concurrent participation in other trials. The study was approved by the Ethics Committee of Shanghai Children’s Medical Center (SCMCIRB-K2023015-1). Written informed consent was obtained from all legal guardians.

### Pharmacotherapeutic regimen

All patients received ACTH following a standardized protocol [21]. ACTH was initiated at 12.5–25 U/d based on age and weight, with efficacy evaluated every three days. Doses were adjusted based on response and tolerability. Total ACTH duration did not exceed four weeks, followed by oral prednisone taper. The ACTH used in this study (specification: 25 U per vial; National Drug Approval Number: H31022101) was manufactured by Shanghai First Biochemical & Pharmaceutical Co., Ltd., Shanghai, China. We also calculated the new ASMs that added during hospitalization, and dose adjustments of existing medications were not included as new ASMs.

### Study population

A total of 209 patients were identified. For children with multiple prior ACTH courses, our study analyzed the most recent ACTH course given during our hospitalization. After applying inclusion criteria, 174 patients met eligibility, and 12 were excluded due to insufficient data (*n* = 4), ACTH allergy (*n* = 1), tuberous sclerosis (*n* = 5), or post‑transplant immunosuppression (*n* = 2). Finally, 162 patients were included.

### Definition of treatment response

Response was assessed at three-day intervals. Outcomes were categorized as [22]: complete remission (absence of spasms with EEG normalization); responders (≥ 50% spasm reduction); and non-responders (< 50% reduction or persistent hypsarrhythmia despite ≥ 50% seizure reduction). Short-term response was defined as the response measured at the completion of the current ACTH course, with an allowable assessment window of ± 2 days. For analysis, response was dichotomized as responder (≥ 50% reduction) versus non-responder. The overall ACTH therapeutic response rate was calculated as: (Number of patients with complete remission + Number of responders) / Total number of cases × 100%.

### Data collection

Data encompassed three domains: (1) Demographic and clinical characteristics: age, sex, gestational age, perinatal history, age at spasm onset, EEG findings (hypsarrhythmia), brain MRI, genetic results, developmental status; (2) Pre-treatment history: prior ASMs (types, numbers), prior steroid/ACTH use, prior vigabatrin/ketogenic diet, interval from onset to ACTH; and (3) On-treatment events: ASMs at ACTH initiation, medications added/discontinued during therapy, ACTH duration, frequency of dose adjustments.

### Statistical analysis

Statistical analyses were conducted using SPSS (Version 26; IBM Corp.). To eliminate confounding effects from baseline differences between groups, propensity score matching (PSM) was performed [23]. Propensity scores were calculated using logistic regression with treatment response as the outcome variable. Covariates were selected based on clinical relevance and variables with *P* < 0.20 in pre-matching univariate analysis. Patients were matched 1:1 using a nearest-neighbor algorithm without replacement, with a caliper width of 0.2 times the standard deviation of the propensity score logit [23]. Standardized differences < 0.10 indicated adequate balance. Continuous variables were compared using t-tests or Mann-Whitney U tests, and categorical variables using Chi-square or Fisher’s exact tests. After PSM, univariate analysis identified variables associated with response (*P* < 0.20), which were entered into penalized logistic regression with L1 penalty (LASSO). The outcome was defined as good short-term response (1 = yes, 0 = no). OR < 1 indicated lower odds of good response (poor prognostic factor), while OR > 1 indicated higher odds of good response. LASSO was used to mitigate small-sample bias and unstable estimates in sparse subgroups. Odds ratios (OR) and 95% confidence intervals (CI) were calculated, with *P* < 0.05 considered significant.

## Results

### Patient characteristics before and after PSM

**Fig. 1:**
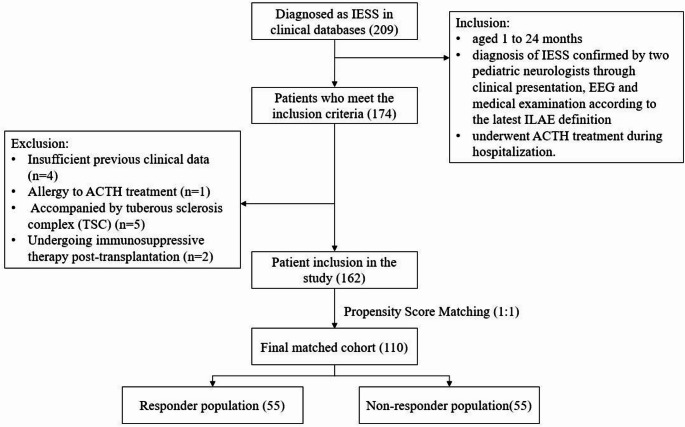
Flow diagram of infants with Epileptic Spasms Syndrome who met the inclusion and exclusion criteria in the study

A total of 162 infants were enrolled (Fig. 1). Overall short-term response rate was 54.3% (88/162). Before PSM, responders and non-responders differed significantly in age at spasm onset (*P* = 0.006), with responders more likely to have onset at 4–12 months (76.1% vs. 52.7%). No other baseline differences were observed (Table 1). After 1:1 PSM using covariates with *P* < 0.20, 110 patients (55 responders, 55 non-responders) were included, with all baseline characteristics well balanced (standardized differences < 0.10) (Table 1). Among the 162 patients, 111 (68.5%) had developmental abnormalities. Various abnormalities on brain MRI were found in 121 of 162 patients (74.7%). A total of 80 patients (49.4%) underwent genetic testing (whole-exome sequencing), of whom 19 (23.8%) had identifiable genetic abnormalities (Table 1s).

**Table 1:** Baseline characteristics of responder and non-responder groups

Characteristics	Before PSM		*P* value	After PSM		*P* value
	Responder population (*n* = 88)	Non-responder Population (*n* = 74)		Responder population (*n* = 55)	Non-responder Population (*n* = 55)	
Sex, male/female	54/34	42/32	0.552	33/22	33/22	1.000
Age, (range), months	13.69 ± 14.29 (3.17–86.33)	17.23 ± 20.25 (1.60-121.90)	0.195	14.54 ± 11.71 (3.17–66.93)	13.86 ± 9.44 (2.73–46.43)	0.867
Onset age of spasms (%), months			0.006			0.975
≤ 3	11 (12.5)	22 (29.7)		10 (18.2)	11 (20.0)	
4–12	67 (76.1)	39 (52.7)		36 (65.4)	34 (63.6)	
13–24	10 (11.4)	13 (17.6)		9 (16.4)	12 (16.4)	
Preterm infant at<37 weeks (%)	9 (12.2)	9 (10.2)	0.696	4 (7.3)	8 (14.5)	0.359
Neonatal Brain Injury History (%)	26 (29.5)	26 (35.1)	0.448	17 (30.9)	19 (34.5)	0.684
Growth and Developmental Delay (%)	61 (69.3)	50 (67.6)	0.811	40 (72.7)	37 (67.3)	0.533
Hypsarrhythmia on VEEG (%)	38 (43.2)	35 (47.3)	0.600	23 (41.8)	25 (45.5)	0.701
Abnormal Genetic Finding (%)	7 (8.0)	12 (16.20)	0.236	5 (9.1)	7 (12.7)	0.293
Abnormal Brain MRI (%)	63 (71.6)	48 (64.9)	0.654	40 (72.7)	38 (69.1)	0.759

PSM, propensity score matching, VEEG, video electroencephalography, MRI, magnetic resonance imaging

### Association between pre-treatment history and ACTH response

**Fig. 2:**
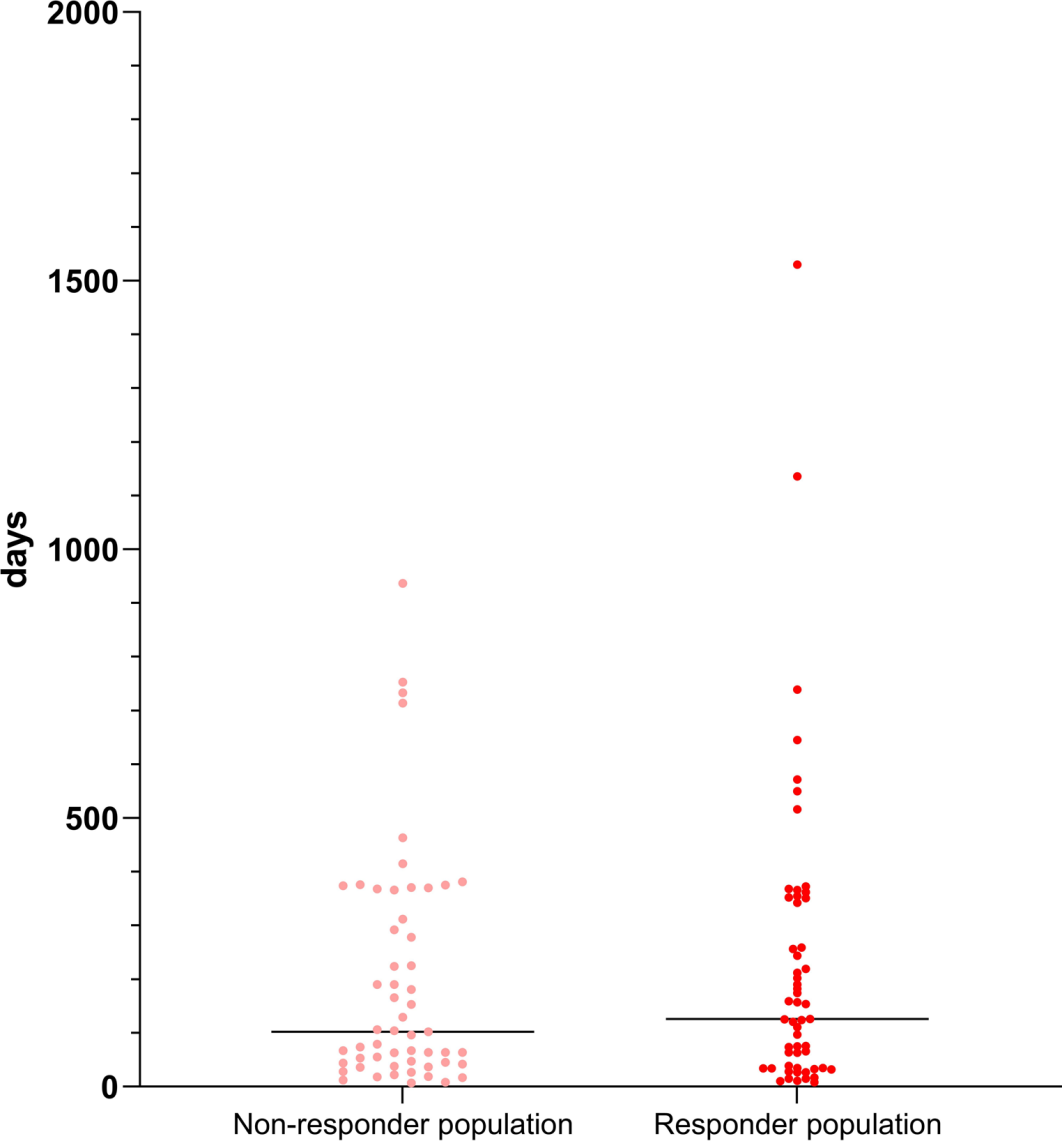
Distribution of time from spasms onset to the current adrenocorticotropic hormone treatment between responder and non-responder groups after PSM

To determine whether pre‑treatment history influenced short‑term response, we compared the distribution of pre‑treatment variables between responder and non‑responder groups in the matched cohort (Table 2). The time interval of spasm onset and current ACTH intervention really varied. Patients treated within 15 days had a higher short term response rate (62.5%) than those with delayed treatment (49.0%), which meant shorter untreated spasm duration predicts better response to hormonal therapy. The exact onset-to-current treatment interval (in days) for each patient was shown in Fig. 2. No significant correlation was detected between the number of prior ASMs and the onset to treatment interval in the cohort. The number of prior ASMs was significantly associated with response (*P* = 0.045). Patients with 4–7 prior ASMs showed uniformly poor response (0/5), whereas those with no prior ASMs had a response rate of 42.9% (9/21) and those with 1–3 prior ASMs achieved 54.8% (46/84). Prior steroid/ACTH use exhibited a trend toward higher response (70.6% vs. 46.2%, *P* = 0.065). Prior vigabatrin or ketogenic diet use, interval from onset to ACTH, and prior intervention were not significantly associated.

**Table 2:** Differences in treatment history before hospitalization between responder and non-responder groups after PSM

Index	Sample, *n*	Responder population(*n* = 55)	Non-responder Population (*n* = 55)	*P* value
Prior intervention for IESS (%)	89	46 (51.7)	43 (48.3)	0.467
T_ot_ (%), days				0.716
≤ 15	8	5 (62.5)	3 (37.5)	
> 15	102	50 (49.0)	52 (51.0)	
Number of prior ASMs used^1^ (%)				0.045
0	21	9 (42.9)	12 (57.1)	
1 ~ 3	84	46 (54.8)	38 (45.2)	
4 ~ 7	5	0 (0.0)	5 (100.0)	
Prior steroid/ACTH used (%)	17	12 (70.6)	5 (29.4)	0.065
Prior vigabatrin used (%)	7	3 (42.9)	4 (57.1)	1.000
Prior ketogenic diet used (%)	7	3 (42.9)	4 (57.1)	1.000

ACTH, adrenocorticotropic hormone, PSM, propensity score matching, IESS, infantile epileptic spasms syndrome, T_ot_, time from spasms onset to adrenocorticotropic hormone treatment, ASMs, antiseizure medications

¹Number of distinct antiseizure medication types based on generic name

### On‑treatment events as early indicators of short-term response

We then examined whether clinical decisions made during ACTH therapy could signal subsequent response (Table 3). The addition of new ASMs during therapy was strongly associated with poor response (*P* < 0.001): only 20.0% (6/30) of those requiring additions responded, compared to 61.3% (49/80) without additions. The frequency of ACTH dose adjustments also showed a significant inverse relationship with response (*P* = 0.010). Response rates declined progressively with more adjustments: 73.3% with no adjustments, 59.1% with one adjustment, and 35.3% with two or more adjustments. Other events, including the number of ASMs at ACTH initiation, discontinuation of ASMs, concomitant vigabatrin or ketogenic diet, and ACTH treatment duration, were not significantly associated.

**Table 3:** Comparison of clinical events during hospitalization between responder and non-responder groups after PSM

Index	Sample, *n*	Responder population(*n* = 55)	Non-responder Population (*n* = 55)	*P* value
Number of ASMs at ACTH initiation^1^ (%), days				0.082
0	16	10 (62.5)	6 (37.5)	
1 ~ 3	90	45 (50.0)	45 (50.0)	
4 ~ 5	4	0 (0.0)	4 (100.0)	
Addition of new ASMs during therapy (%)	30	6 (20.0)	24 (80.0)	**< **0.001
Discontinuation of ASMs during therapy (%)	8	5 (62.5)	3 (62.5)	0.716
Vigabatrin use during therapy (%)	20	10 (50.0)	10 (50.0)	1.000
Ketogenic diet use during therapy (%)	11	3 (27.3)	8 (72.7)	0.202
ACTH treatment duration (%), days				0.756
≤ 7	10	6 (60.0)	4 (40.0)	
8–14	75	36 (48.0)	39 (52.0)	
> 14	25	13 (52.0)	12 (48.0)	
Frequency of ACTH dose adjustments (%)				0.010
0	15	11 (73.3)	4 (26.7)	
1	44	26 (59.1)	18 (40.9)	
≥ 2	51	18 (35.3)	33 (64.7)	

ACTH, adrenocorticotropic hormone, PSM, propensity score matching, ASMs, antiseizure medications

¹Number of distinct antiseizure medication types based on generic name

### Multivariate analysis of factors associated with good short-term ACTH response

To identify independent predictors of poor response, we entered variables with *P* < 0.20 in univariate analysis (number of prior ASMs, prior steroid/ACTH use, number of ASMs at ACTH initiation, addition of new ASMs, and frequency of dose adjustments) into penalized logistic regression (LASSO) (Table 4). The outcome was good short-term response (1 = yes, 0 = no). This model addressed unstable estimates caused by very small subgroups (e.g., *n* = 5 patients with 4–7 prior ASMs, *n* = 4 patients with 4–5 ASMs at initiation, all non-responders). After adjustment, addition of new ASMs during therapy (OR = 0.055, 95% CI: 0.012–0.250, *P* < 0.001) and frequency of dose adjustments (1 adjustment: OR = 0.153, 95% CI: 0.030–0.780, *P* = 0.024; ≥2 adjustments: OR = 0.251, 95% CI: 0.086–0.731, *P* = 0.012) had strong associations with lower odds of good response. Prior steroid/ACTH use showed a trend toward higher odds of good respond (OR = 2.941, *P* = 0.096). Weak predictors were shrunk to zero by LASSO, indicating no independent association. Notably, these factors likely represent markers of refractory disease, not independent early predictors of response.

**Table 4:** Penalized logistic regression of independent indicators associated with short-term ACTH response

variable	β	OR	95% CI	*P* value
Number of prior ASMs used^1^				
0^*^		1.000		
1 ~ 3	0.292	1.338	0.509–3.531	0.553
4 ~ 7	-1.683	0.185	0.030–1.104	0.067
Prior steroid/ACTH use before hospitalization	1.079	2.941	0.813–10.610	0.096
Number of ASMs at ACTH initiation^1^				
0^*^		1.000		
1 ~ 3	0.183	1.201	0.428–3.373	0.722
4 ~ 5	0.000	1.000	-	0.999
Addition of new ASMs during therapy	-2.892	0.055	0.012–0.250	<0.001
Frequency of ACTH dose adjustments				
0^*^		1.000		
1	-1.872	0.153	0.030–0.780	0.024
≥ 2	-1.380	0.251	0.086–0.731	0.012

ACTH, adrenocorticotropic hormone; OR, odds ratio; CI, confidence interval; ASMs, antiseizure medications

¹Number of distinct antiseizure medication types based on generic name

^*^Reference category

Outcome was defined as short-term ACTH response (1 = good response, 0 = poor response). LASSO penalized regression was used to mitigate small-sample bias and unstable estimates. Variables with OR = 1.000 were shrunk to zero, indicating no independent predictive value

## Discussion

This propensity score‑matched study demonstrates that particular on‑treatment events during ACTH therapy, specifically the addition of new ASMs and frequent dose adjustments, may correlate with reduced likelihoods of favorable short-term outcomes in IESS, although confidence intervals are broad. Notably, these factors are not independent early predictors but likely represent markers of refractory disease, as they arise from therapeutic adjustments made when ACTH fails to elicit an early response.

A Class I randomized controlled trial has demonstrated that low-dose (20–30 IU/day) ACTH is probably as effective as high-dose (150 IU/m²) for short-term treatment, with comparable spasm cessation and hypsarrhythmia resolution [10]. Given that higher ACTH doses are associated with increased risks of hypertension, irritability and infection, our individualized adjustment strategy aims to optimize efficacy while minimizing adverse effects. The overall response rate of 54.3% in our cohort is consistent with previous reports ranging from 36.7% to 87% [7–9]. A recent study involving 532 children reported that only 160 achieved initial response to first‑line treatment, underscoring the difficulty of achieving spasm control [19]. Another study of 88 IESS patients found a 53.4% response rate to ACTH, with a relapse rate of 42.6% among responders [17]. Our findings corroborate these real‑world challenges and highlight the need for early identification of patients likely to fail ACTH monotherapy. The most clinically relevant finding is that on‑treatment events can serve as early warning signals. The addition of new ASMs during ACTH therapy was strongly associated with treatment failure in both univariate and multivariate analyses. Rather than implying causation, this event merely reflects the clinician’s perception of insufficient efficacy-a common trigger for adding medications in practice [24]. Therefore, the initiation of a new ASM should prompt immediate reassessment of the therapeutic strategy, rather than simply continuing to escalate polypharmacy [20].

Similarly, the frequency of ACTH dose adjustments emerged as an independent indicator of poor response. Patients requiring no adjustments had the highest response rate (73.3%), while those needing two or more adjustments fared worst (35.3%). This suggests that patients who need multiple dose modifications are inherently less responsive to standard ACTH dosing. Previous studies have shown that the response to ACTH is usually determined early in the course [25], and that a reduction in BASED score by ≥ 2 at day 14 may signal a favorable outcome [18]. Our results extend these observations by demonstrating that frequent dose adjustments should alert clinicians to consider alternative or combination approaches.

In univariate analysis, a higher number of prior ASMs was associated with poorer response, consistent with the notion that prior ASM exposure may reflect more refractory epilepsy [17, 18]. However, neither the number of prior ASMs nor the number of ASMs at ACTH initiation retained statistical significance in the multivariate model. This is likely explained by the small numbers of patients in the highest exposure categories: five patients had used 4–7 prior ASMs at ACTH initiation, all of whom were non‑responders. The limited sample sizes in these subgroups restricted statistical power to detect independent effects. According to international standard treatment (UKISS/ICISS), hormonal therapy or vigabatrin is recommended as first‑line for IESS. However, in our real-world cohort, some patients even had received 4‑7 ASMs prior to ACTH. This largely reflects referral delays and the fact that prior treatments at primary or secondary hospitals were not always standardized, often due to practical constraints such as patient volume, medication availability, and consultation time. This also highlights the gap between clinical trial evidence and routine practice.

Despite the lack of statistical significance, these findings carry important clinical implications. Patients with extensive prior ASM exposure (4–7 medications) and those on multiple ASMs (4–5 medications) at ACTH initiation represent a highly refractory population, as evidenced by their uniformly poor response. For such patients, the likelihood of response to ACTH monotherapy appears extremely low, suggesting that repeated ACTH courses may be of limited benefit and that earlier consideration of alternative approaches such as vigabatrin, ketogenic diet, or epilepsy surgery may be more appropriate [19, 20]. In contrast, prior steroid or ACTH use showed a trend toward better response (OR = 3.203, *P* = 0.102), suggesting that previous hormone exposure does not preclude subsequent response and may even be advantageous in selected patients [18]. The clinical value of these indicators lies in their ability to guide timely treatment modifications. Rather than persisting with potentially ineffective ACTH monotherapy while sequentially adding drugs or repeatedly adjusting doses, clinicians who recognize these signals can promptly transition to alternative first‑line agents (e.g., vigabatrin) or consider non‑pharmacological options such as ketogenic diet or epilepsy surgery [26]. This approach aligns with the “treat early and treat effectively” paradigm, which has been shown to improve outcomes in IESS [19, 27].

From a biological perspective, the need for additional ASMs or frequent dose adjustments may reflect underlying epileptogenic processes that are less responsive to ACTH‑mediated mechanisms. ACTH exerts its effects through cortisol release, direct modulation of melanocortin receptors, and potential neurotrophic actions [28]. Patients with cortical dysplasia, genetic mutations affecting synaptic function, or other structural abnormalities may have disease that is inherently less hormone‑sensitive [4, 14]. The uniformly poor response observed in patients with extensive prior polypharmacy may reflect a more severe underlying epileptogenic substrate that is unlikely to respond to hormonal therapy alone. Future studies incorporating detailed etiological characterization, including advanced neuroimaging and genetic testing, could help elucidate the biological basis of these observations.

In our study, the use of propensity score matching with covariates selected based on pre‑matching analysis (*P* < 0.20) minimized confounding and ensured comparability between responder and non‑responder groups. Nevertheless, several limitations warrant careful consideration. First, the retrospective design inherently introduces selection and information biases, and although PSM balanced measured confounders, other factors influencing clinicians’ threshold for adding ASMs or adjusting doses could not be ruled out. Second, the modest sample size after matching, particularly in small subgroups (e.g., only 5 patients with 4–7 prior ASMs), limited statistical power for analyses, as reflected in wide confidence intervals. This highlights the need for larger multicenter studies to validate these findings. Third, the original logistic regression produced unstable estimates in small subgroups, which we addressed using penalized logistic regression (LASSO) to improve robustness. Furthermore, new ASM addition and frequent dose adjustments should not be overinterpreted as causal; rather, they likely serve as clinical markers of refractory disease, reflecting real-world decisions made when the initial ACTH response is judged suboptimal. This distinction is critical because these events are not independent early prognosticators but signals of a failing strategy. Fourth, our outcome assessment was restricted to the ACTH treatment period, long‑term outcomes were not evaluated. Fifth, genetic testing was not universally performed owing to the retrospective nature of this study. Although genetic findings were balanced between groups after PSM, the possibility of residual bias due to the limited sample size and incomplete testing could not be entirely excluded. Despite the above limitations, our findings provide practical value for clinicians by identifying readily observable signals that can assist real‑time treatment decisions.

## Conclusions

The addition of ASMs and repeated ACTH dose adjustments during therapy likely reflect early clinical concern for suboptimal response and should prompt timely reassessment of treatment strategy. Patients with extensive prior ASM exposure or those requiring multiple ASMs at treatment initiation represent a highly refractory subgroup in whom repeat ACTH therapy may be of limited benefit. Recognizing these signals and timely adjusting treatment strategy may improve outcomes. Future research should integrate these observable signals into clinical decision-making algorithms and validate them in larger, prospective cohorts.

## Supplementary Information

Below is the link to the electronic supplementary material.

Supplementary Material 1.

## Data Availability

The datasets are available from the corresponding author on reasonable request.

## Abbreviations

ACTH: Adrenocorticotropic hormone
ASM: Antiseizure medication
BASED: Burden of Amplitudes and Epileptiform Discharges
CI: Confidence interval
EEG: Electroencephalography
IESS: Infantile epileptic spasms syndrome
ILAE: International League Against Epilepsy
LASSO: Logistic regression with L1 penalty
MRI: Magnetic resonance imaging
OR: Odds ratio
PSM: Propensity score matching
STROBE: Strengthening the Reporting of Observational Studies in Epidemiology
