# Supplementary material for: Treatment history as early indicators of short-term response to ACTH therapy in infantile epileptic spasms syndrome: a propensity score matching analysis
Source: Neurol Res Pract. 2026 Jul 14;8(1):55. doi: 10.1186/s42466-026-00513-4 (PMC13366787; doi:10.1186/s42466-026-00513-4)
Supplement: Supplementary file 1 — Supplementary Material 1. [file 42466_2026_513_MOESM1_ESM.docx]

**Table 1S.** Baseline characteristics of responder and non-responder groups.

| Characteristics | Before PSM | | *P* value | After PSM | | *P* value |
| --- | --- | --- | --- | --- | --- | --- |
|  | Responder population  (*n* = 88) | Non-responder  Population  (*n* = 74) |  | Responder population  (*n* = 55) | Non-responder  Population  (*n* = 55) |  |
| Development before treatment (%) |  |  | 0.811 |  |  | 0.533 |
| Roughly normal | 27 (30.7) | 24 (32.4) |  | 15 (67.3) | 18 (32.7) |  |
| Delayed development | 61 (69.3) | 50 (67.6) |  | 40 (72.7) | 37 (48.1) |  |
| Hypsarrhythmia on VEEG (%) |  |  | 0.600 |  |  | 0.701 |
| Yes | 38 (43.2) | 35 (47.3) |  | 23 (41.8) | 25 (45.5) |  |
| No | 50 (56.8) | 39 (52.7) |  | 32 (58.2) | 30 (54.5) |  |
| Genetic Finding (%) |  |  | 0.236 |  |  | 0.293 |
| Normal | 36 (40.9) | 25 (33.8) |  | 26 (47.3) | 18 (32.7) |  |
| Abnormal | 7 (8.0) | 12 (16.2) |  | 5 (9.1) | 7 (12.7) |  |
| Unknown | 45 (51.1) | 37 (50.0) |  | 24 (43.6) | 30 (54.6) |  |
| Brain MRI (%) |  |  | 0.654 |  |  | 0.759 |
| Normal | 20 (22.7) | 21 (28.4) |  | 12 (21.8) | 12 (21.8) |  |
| Abnormal | 63 (71.6) | 48 (64.9) |  | 40 (72.7) | 38 (69.1) |  |
| Unknown | 5 (5.7) | 5 (6.8) |  | 3 (5.5) | 5 (9.1) |  |

Abbreviations: PSM, propensity score matching, VEEG, video electroencephalography, MRI, magnetic resonance imaging.
